# Acute Myocardial Infarction and Massive Pulmonary Embolus Presenting as Cardiac Arrest: Initial Rhythm as a Diagnostic Clue

**DOI:** 10.1155/2013/343918

**Published:** 2013-07-14

**Authors:** Nirmanmoh Bhatia, Haree Vongooru, Sohail Ikram

**Affiliations:** ^1^Department of Medicine, University of Louisville, Louisville, KY 40202, USA; ^2^Division of Cardiovascular Medicine, Department of Medicine, University of Louisville, Louisville, KY 40202, USA

## Abstract

Myocardial infarction (MI) and massive pulmonary embolism (MPE) are common causes of cardiac arrest. We present two cases with similar clinical presentation and EKG findings but different initial rhythms. *Case  1*. A 55-year-old African American male (AAM) was brought to the emergency room (ER) with cardiac arrest and pulseless electrical activity (PEA). Twelve-lead electrocardiogram (EKG) was suggestive of ST segment elevations (STEs) in anterolateral leads. Coronary angiogram did not reveal any significant obstruction. An echocardiogram was suggestive of a pulmonary embolus (PE). Autopsy revealed a saddle PE. *Case  2*. A 45-year-old AAM with a history of coronary artery disease was brought to the ER after ventricular fibrillation (VF) arrest. Twelve-lead EKG was suggestive of STE in anterior leads. Coronary angiogram revealed in-stent thrombosis. In cardiac arrests, distinguishing the two major etiologies (MI and MPE) can be challenging. PEA is more commonly associated with MPE versus MI due to near complete obstruction of pulmonary blood flow with an intact electrical conduction system. MI is more commonly associated with VF as the electrical conduction system is affected more often by ischemia. In conclusion, the previous cases illustrate that initial rhythm may be a vital diagnostic clue.

## 1. Background

Myocardial infarction (MI) and massive pulmonary embolism (MPE) are common causes of cardiopulmonary arrest and together constitute about two-thirds of out-of-hospital arrests of no immediate apparent cause [1]. These entities may present with similar clinical features of chest pain, shortness of breath, hemodynamic instability, and cardiac arrest. Electrocardiograms (EKGs) may have striking similarities in these cases, sometimes not leading to the correct diagnosis [2, 3]. An important clue to differentiate the two could be the initial rhythm.

 We present two cases with similar clinical presentation and EKG changes but completely different etiology. An important diagnostic clue was the rhythm at presentation.

## 2. Case Presentations

**Case 1**

**Figure 1:**
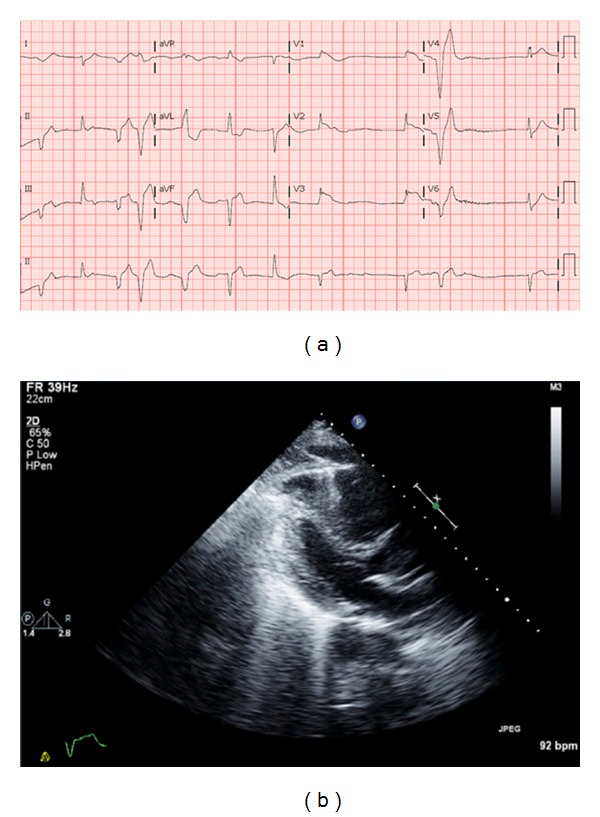
12-lead EKG showing frequent premature ventricular complexes (PVC), complete right bundle branch block, and ST segment elevation in anterior leads. Parasternal long axis view showing severe RV volume and pressure overload.

A 55-year-old African American male was brought to the emergency room (ER) after a witnessed syncopal episode on the street. He was nasally intubated and resuscitated for 15 minutes after which he regained a pulse. In the ER, the patient lost his pulse again and went into pulseless electrical activity (PEA). Advanced cardiac life support (ACLS) protocol was initiated, and attempts at resuscitation were eventually successful after multiple cycles of cardiopulmonary resuscitation (CPR). A 12-lead EKG after regaining pulse was consistent with sinus tachycardia, right bundle branch block, and ST segment elevations in anterior and lateral leads (Figure 1). He was taken to the cardiac catheterization laboratory for emergent coronary angiography, which showed no significant coronary artery disease (CAD), and all vessels had thrombolysis in myocardial infarction (TIMI) III flow. An immediate transthoracic echocardiogram (TTE) revealed normal left ventricular function, right ventricular (RV) pressure overload, RV dyskinesia, and a flattened interventricular septum (Figure 1). This was highly suspicious for an acute pulmonary embolus (PE). The patient could not be administered thrombolytics as he had started bleeding from his nose and arteriotomy site during the catheterization. He remained hemodynamically unstable and was not deemed a candidate for surgical or catheter based thrombectomy. He passed away in less than 2 hours after sustaining multiple episodes of ventricular tachycardia (VT) that degenerated into ventricular fibrillation (VF) arrest. An autopsy revealed a saddle pulmonary embolus.

**Case 2**

**Figure 2:**
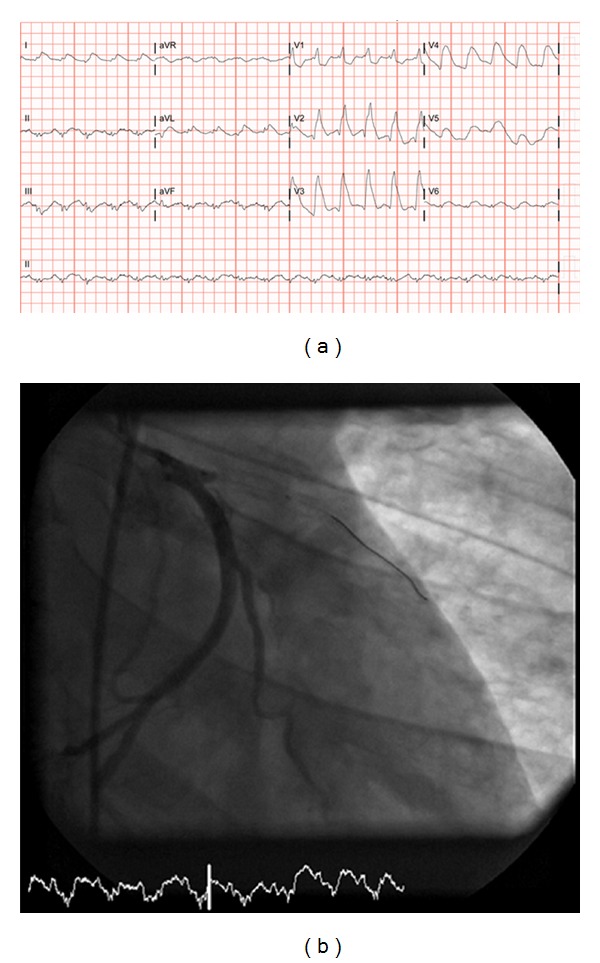
12-lead EKG showing sinus tachycardia, complete right bundle branch block, and massive ST segment elevation in anterior, anterolateral, and lateral leads. Coronary angiography after balloon angioplasty for stent thrombosis showing no reflow phenomenon, suggesting emboli to microvasculature and possibly nonviable myocardium with interstitial or perivascular myocardial edema.

A 45-year-old African American male with a history of CAD and coronary stenting had a severe episode of chest pain on the day of presentation that prompted him to visit the ER. En route, he lost consciousness, and his vehicle stopped against a sidewall without any major physical damage. Emergency medical service (EMS) found the patient in VF. He was successfully resuscitated and transferred to ER. The patient subsequently had another episode of VF arrest. He was resuscitated multiple times. Spontaneous circulation was established after 60 minutes of CPR and 14 attempts at defibrillation. His 12 lead EKG is shown (Figure 2). Transthoracic echocardiogram in the ER showed apical dyskinesis, septal akinesis, and severe hypokinesis of anterior and lateral walls with a left ventricular ejection fraction of 35%. It was decided not to proceed with emergent coronary angiography given the prolonged resuscitation time and subsequent comatose state. After 12 hours, his mental status improved and coronary angiography revealed stent thrombosis in proximal left anterior descending artery (LAD). There was TIMI I flow in mid-distal LAD (Figure 2). Aspiration thrombectomy was performed followed by balloon angioplasty, and no improvement was noted in the flow in LAD. His clinical status deteriorated, and with no reasonable hope for survival, his family decided to withdraw care.

## 3. Discussion

The previous cases highlight the importance of considering the initial rhythm in patients presenting with cardiac arrest before committing to a diagnosis of MI or PE based on other clinical and objective parameters. When patients are hemodynamically stable, imaging modalities like computed tomography and pulmonary angiography play a crucial role in establishing a diagnosis of PE and differentiating this from MI. However, this may not be feasible in critically ill patients. Pulmonary emboli resulting in cardiac arrest have a very poor prognosis [4]. 

PEA has been associated with significant hypoxia, profound acidosis, severe hypovolemia, tension pneumothorax, electrolyte imbalance, drug overdose, sepsis, large MI, MPE, cardiac tamponade, hypoglycemia, hypothermia, and trauma [5]. In Case 1, all of the previous causes of PEA except MPE and MI were excluded. 

PEA has been defined as myocardial electrical activity without associated mechanical activity [6]. This pathophysiology gives a vital insight into the presence of this rhythm in cases of MPE presenting with cardiac arrest. With a MPE, the blood flow to the left side of the heart is markedly diminished. The electrical system of the heart continues to function normally. As a result, cardiac output is drastically reduced, impairing the systemic and coronary circulation.

In case of an MI, especially STEMI, the conduction system may be affected initially, leading to arrhythmias [7]. Inferior wall MI may cause bradyarrhythmias, RV infarction can lead to a complete heart block, and anterior wall MI can give rise to infranodal rhythms [8].

Hence, it is not surprising that PEA is commonly associated with MPE [9]. Alternatively, acute coronary syndrome (ACS) is more likely to present with VT or VF arrest [10]. The initial rhythm may therefore lead to a diagnosis in cases of cardiac arrest where ischemia is being strongly considered. This has the potential for improving outcomes.

Since PEA is the presenting rhythm in 63% of PE induced cardiac arrests, echocardiography may provide critical information to differentiate between MI and PE in cardiac arrests with PEA. Further, some guidelines have recommended early use of this modality to establish a diagnosis in cases of suspected pulmonary embolism [11].

In cardiac arrests with ongoing cardiac activity, regional wall motion abnormalities (RWMA) may be detected in cases of new ischemia or previous infarctions [12]. Although, this may not establish an absolute diagnosis, an ischemic etiology should be suspected if the RWMA correspond with EKG evidence of ischemia in the same region and an early coronary intervention may be performed.

Alternatively, RV dilatation in the absence of a left-sided pathology—valvular or ventricular disease or established pulmonary disease—would be suggestive of a PE [12]. McConnell's sign (regional wall motion abnormalities sparing the right ventricular apex), if present, has a 94% specificity for a PE [13].

## 4. Conclusion

Initial rhythm—PEA or VF may be a vital clue in differentiating MI and MPE as the underlying etiology in patients presenting with cardiac arrest. This in turn can lead to early initiation of appropriate therapy leading to better outcomes. In cardiac arrests with PEA and EKG changes suspicious of a MI, performing echocardiography before coronary angiography may improve final outcomes.
